# Drp1–associated genes implicated in sepsis survival

**DOI:** 10.3389/fimmu.2024.1516145

**Published:** 2025-01-08

**Authors:** Marissa D. Pokharel, Anlin Feng, Ying Liang, Wenli Ma, Saurabh Aggarwal, Hoshang Unwalla, Stephen M. Black, Ting Wang

**Affiliations:** ^1^ Center for Translational Science, Florida International University, Port Saint Lucie, FL, United States; ^2^ Department of Cellular and Molecular Medicine, Florida International University, Miami, FL, United States; ^3^ Department of Environmental Health Sciences, Florida International University, Miami, FL, United States

**Keywords:** DRP1, fission, mitochondria, sepsis survival, inflammation

## Abstract

Sepsis is a severe and life-threatening medical syndrome that can lead to organ failure and death. Despite advances in medical treatment, current therapies are often inadequate, with high septic mortality rates. Therefore, there is a critical need for reliable prognostic markers to be used in clinical settings to improve the management and outcomes of patients with sepsis. Recent studies have suggested that mitochondrial dynamics, including the processes of mitochondrial fission and fusion, are closely related to the severity of sepsis and the status of inflammation. By monitoring transcriptomic signals related to mitochondrial dynamics, new and reliable biomarkers can be engineered to more accurately predict sepsis survival risk. Such biomarkers would be invaluable in clinical settings, aiding healthcare providers in the early identification of high-risk patients and improving treatment strategies. To achieve this goal, we utilized the major mitochondrial fission regulatory protein dynamin-related protein 1 (Drp1, gene code *DNM1L*) and identified Drp1-associated genes that are enriched with sepsis survival genes. A 12-gene signature (GS) was established as a differentially expressed gene (DEG)-based GS. Next, we compared genes of proteins that interact with Drp1 to sepsis survival genes and identified 7 common genes, establishing a GS we term as protein-protein interaction (PPI)-based GS. To evaluate if these GSs can predict sepsis survival, we used publicly available human blood transcriptomic datasets from sepsis patients. We confirmed that both GSs can successfully predict sepsis survival in both discovery and validation cohorts with high sensitivity and specificity, with the PPI-based GS showing enhanced prognostic performance. Together, this study successfully engineers a new and validated blood-borne biomarker (PPI-based 7-gene GS) for sepsis survival risk prediction. This biomarker holds the potential for improving the early identification of high-risk sepsis patients and optimizing personalized treatment strategies to reduce sepsis mortality.

## 1 Introduction

Sepsis is a deadly syndrome comprised of many dysregulated pathways in response to infection or injury, ultimately leading to organ dysfunction and damage (1). Sepsis remains a major public health concern due to its high incidence, mortality rate, and strain on the hospital care system (2). The mortality rate is estimated to be 41.9% for intensive care unit (ICU) septic patients (3). Unfortunately, increasing sepsis survival is not the only hurdle in treating sepsis; but quality of life after sepsis is a major concern (4–6). It is estimated that nearly 75% of sepsis survivors have deficits in at least one activity of daily life (7). Currently, the only treatment for sepsis is the administration of intravenous broad-spectrum antibiotics, with the ideal administration within an hour of sepsis recognition (8). Sepsis survival drops 7.6% every hour that administration of antibiotics is delayed (9). This short window for antibiotic administration means early detection of sepsis is critical.

Sepsis treatment includes supportive care, which involves the initiation of vasopressors and aggressive fluid therapy (10, 11). However, supportive care only offers limited/modest success (12, 13). This means that the only proven treatment to reduce sepsis mortalities is early detection and immediate administration of antibiotics. Unfortunately, there are no reliable biomarkers or metrics that have been proven to detect sepsis early. Therefore, the only recourse for clinicians is to perform repeated blood cultures to monitor serum lactic acid levels and blood pressure in the hope of identifying sepsis or septic shock cases early (8), causing significant strain on hospital resources. Septic shock is the most severe form of sepsis and patients with septic shock have dire metabolic and cellular abnormalities that are associated with higher mortality rates than sepsis alone (1).

Historically, sepsis progression has been divided into two inflammatory stages. Originally, the first stage was believed to be a pro-inflammatory stage where a cytokine storm induces a profound and intense immune response, followed by a compensatory, anti-inflammatory stage. Early investigations targeted the presumed first, pro-inflammatory stage and evaluated anti-inflammatory treatments. These strategies were unsuccessful in clinical trials (14–16). Newer evidence suggests that sepsis is not biphasic, with a distinct pro-then anti-inflammatory stage, but rather occurs simultaneously (17). This is likely due to the complexity of immune responses, where pathways constantly interact and modulate one another (18). Immunomodulation has been the predominant target tested in sepsis clinical trials. Despite over 100 randomized clinical trials testing immunomodulation, they have failed to yield even one successful drug or strategy (19). This failure likely stems from the cross-talk of the immune response; thus, simply augmenting or inhibiting specific immunomodulators does not have the intended effect as the system responds to this modification. Although further testing is needed to verify why immunomodulation has failed to clinically demonstrate efficacy, the overwhelming failure of immunomodulatory clinical trials emphasizes that this is not a valid strategy for sepsis treatment; therefore, research effort needs to be allocated to other approaches.

Sepsis is known to be the cumulation of multiple dysregulated pathways; therefore, it is critical to identify therapeutics that target multiple pathways (20, 21). Mitochondria are known to play critical roles in a multitude of pathways involved in maintaining homeostasis (22, 23), suggesting that targeting mitochondria may modulate multiple aberrant pathways associated with sepsis progression. For example, mitochondria supply the energy and metabolic intermediates that are needed for immune cell activation and function and influence inflammatory and cell death pathways (24). Dysfunctional mitochondria are well documented in sepsis (25–28), although modulation of mitochondria remains primarily untested. Of importance, mitochondrial dysfunction is evident during the early stages of sepsis and is believed to play a critical role in the initiation of organ damage (29).

Mitochondria are highly dynamic organelles that exist in networks rather than individually. Mitochondrial networks can modulate their function by combining (fusion) or separating (fission). The cell contains a multitude of mitochondrial networks that are constantly performing fusion or fission to modulate their function to adapt to cellular demands, a process referred to as mitochondrial dynamics (23, 30). Since patients with the highest mortality rates have pronounced metabolic and cellular abnormalities (1), it is likely that these patients also exhibit greater mitochondrial dysfunction, although tests are needed to evaluate this postulation. For that reason, we hypothesize that altered mitochondrial function plays a critical role in regulating immune and cell responses, which lead to more severe cases of sepsis and may present an opportunity for earlier diagnosis. Because of the close relationship between mitochondrial dynamics and overall mitochondrial function, our study evaluated dynamin-related protein 1 (Drp1), which is recognized as one of the major regulators of fission and is one of the most studied mitochondrial network mediators (31). Therefore, the aim of this study was first to evaluate the expression of genes related to mitochondrial dynamics to determine if there is a relationship between these genes and sepsis survival. Moreover, we aim to establish a new circulating (Drp1-related) gene signature to predict sepsis survival and validate it as a powerful and independent prognostic tool for further clinical application.

## 2 Materials and methods

### 2.1 Microarray datasets and sepsis survival-related genes

We previously have identified genes that are differentially expressed between patients with a low or high risk of sepsis-related death (32). These genes were identified using two human peripheral blood mononuclear cell datasets from ArrayExpress datasets, E-MTAB-4421 and E-MTAB-4451. Samples from these datasets were collected from patients at 4 weeks after intensive care unit admission. Discovery cohort E-MTAB-4421 includes 187 sepsis survivors and 78 non-survivors, with a male-to-female ratio of 145:120 and a mean age of 62 ± 16 years. Validation cohort E-MTAB-4451 comprises 50 sepsis survivors and 56 non-survivors, with a male-to-female ratio of 79:27 and a mean age of 69 ± 14 years. Differentially expressed genes (DEGs) were only considered significant if the fold change was greater than 1.5 and the false discovery rate (FDR) was less than 5%. Here, we use E-MTAB-4421 as the discovery cohort and E-MTAB-4451 as the validation cohort to test this Drp1-gene signature.

### 2.2 Sepsis survival score

To generate a practical sepsis survival score, we used a linear combination of gene expression values and their corresponding weight values in the Drp1-associated gene signature. The sepsis survival score is as follows:


$$\text{sepsis survival score}=\;\sum_{i=1}^{n}W_{i}\left(\frac{e_{i}-\mu_{i}}{S_{i}}\right)$$

Wherein *n* is the number of genes in the Drp1-associated gene signature for each dataset, *W_i_* shows the weighted value of each gene, *e_i_* is the expression level of each gene, and *μ_i_* and *S_i_* are the mean and standard deviation values, respectively, for the corresponding gene compared the whole sample. Scores above the mean sepsis survival score were classified into the high sepsis survival score cohort, while the values below the mean were classified into the low sepsis survival score cohort.

### 2.3 Enrichment analysis

To generate a list of Drp1-related genes, we first identified genes that significantly changed during Drp1 upregulation using the Gene Expression Omnibus (GEO) dataset GSE182710, referred to as differentially expressed genes (DEGs). To be classified as a DEG, the genes met the following criteria: fold change > 1.5, FRD < 0.05. Here, we refer to these genes as the DEGs-based Drp1-associated genes. Next, we performed a protein-protein interaction (PPI) analysis of the protein, Drp1, to identify proteins that are predicted to interact with Drp1 using the STRING database. The STRING database identifies functional protein association networks. In our analysis, we only used the primary protein interactors (also known as 1^st^ shell interactors), which are proteins that are directly associated with Drp1. Proteins with interaction scores greater than 0.7 were selected and henceforth referred to as PPI-based Drp1-associated genes. The protein network was visualized by Cytoscape (3.10.2).

**Figure 1:**
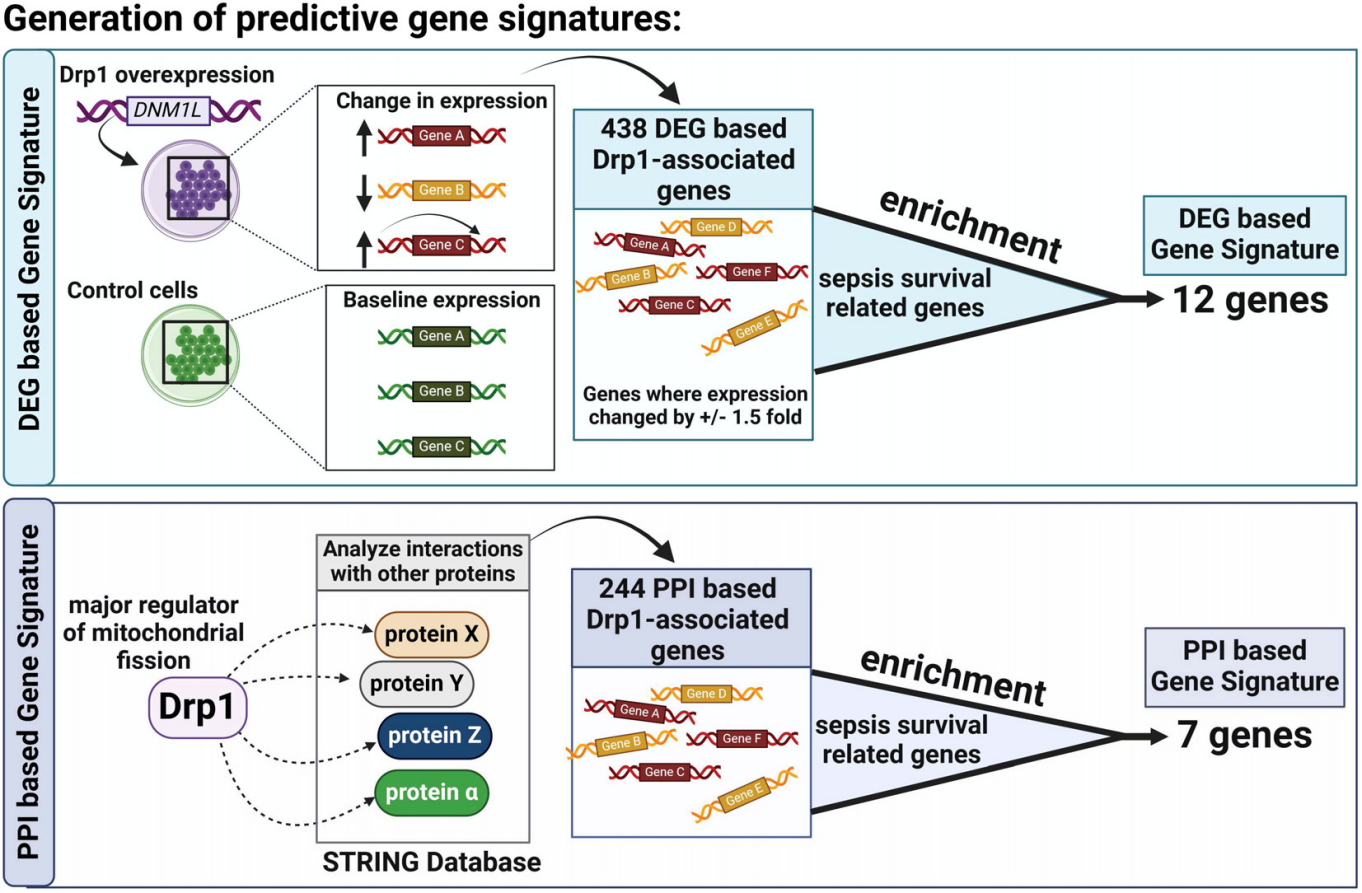
Overview of Gene Signature (GS) Generation. First, we compared Drp1-associated genes to sepsis survival genes and identified 12 common genes, establishing a GS we term as DEG-based GS **(A)**. Next, we compared genes of proteins that interact with Drp1 to sepsis survival genes and identified 7 common genes, establishing a GS we term as PPI-based GS **(B)**. Drp1, Dynamin-related protein 1; DEGs, differentially expressed genes; PPI, protein-protein interaction; GS, gene signature.

The two Drp1-associated gene signatures (GS) were obtained by identifying the common genes between sepsis survival DEGs and the two Drp1-associated genes described above (DEGs-based and PPI-based, **Figure 1**). This enrichment resulted in a 12-gene DEG-based GS and 7-gene PPI-based GS.

Next, we performed Kyoto Encyclopedia of Genes and Genomes (KEGG) analysis of the generated Drp1-associated genes using the database for annotation, visualization, and integrated discovery (DAVID, version 6.8, https://david.ncifcrf.gov/tools.jsp). DAVID is a biological and functional annotation database that aids in the interpretation of gene/signaling pathway interactions. We repeated KEGG analysis for the sepsis-survival genes. We excluded irrelevant pathways, such as diseases that are not associated with sepsis. Adjusted p-values less than 0.05 were considered to be significant.

To validate the accuracy of our PPI analysis, we generated correlation matrixes for both GS using the R package “corrplot” (version 0.92). The genomic annotation file for humans (Genome assembly GRCh37) was obtained from the UCSC Genome Browser. The circular plot was generated by the R package “circlize” (version 0.4.10). All sepsis survival-related genes were visualized as dots, and genes in two gene signatures were marked in the inner circle.

### 2.4 Evaluating prognostic capabilities of GS

To validate that these GSs have the prognostic capability of distinguishing between high and low sepsis risk groups, we performed a variety of assessments. First, we generated gene expression heatmaps of both GS and grouped patients by their sepsis-risk group. The heatmaps were visualized using the R package “gplots” (version 3.1.3.1). The method “complete” was used for hierarchical clustering, and the method “euclidean” was utilized for distance calculation. The survival scores for each GS were calculated and then plotted in violin plots using the R package “ggplot2” (version 3.5.1). The ROC curves were generated by R package “pROC” (version 1.18.5), and the AUC values were calculated by the function “auc”.

### 2.5 Running enrichment score

To determine the running enrichment score, the gene expression matrix of the discovery cohort and gene sets derived from the KEGG pathway were used as input for gene set enrichment analysis (GSEA). The analysis was performed using the default parameters in R package GSEA_R (version 1.2) and adjusted p-values less than 0.25 were considered to be significant. An unsupervised method called Gene Set Variation Analysis (GSVA) by the R package GSVA (version 3.18) was also performed on the discovery cohort and gene sets derived from the KEGG pathway to estimate the variation of KEGG gene sets enrichment across the samples.

### 2.6 CIBERSORT

We estimated the immune cell proportions in the sepsis datasets and evaluated if there were different immune cell proportions between the low- and high-score cohorts generated from the Drp1-PPI GS. We used CIBERSORT (version 1.04) (33) to assess the proportions of immune cell populations in the discovery dataset.

### 2.7 Statistical analysis

The R packages, ade4 (Version: 1.7-22) (34) and pROC (Version: 1.18.5) (35) were used to generate the PCA plots and the ROC curves. Values with false discovery rates (FDR) less than 0.05 were considered to be significant.

## 3 Results

**Figure 2:**
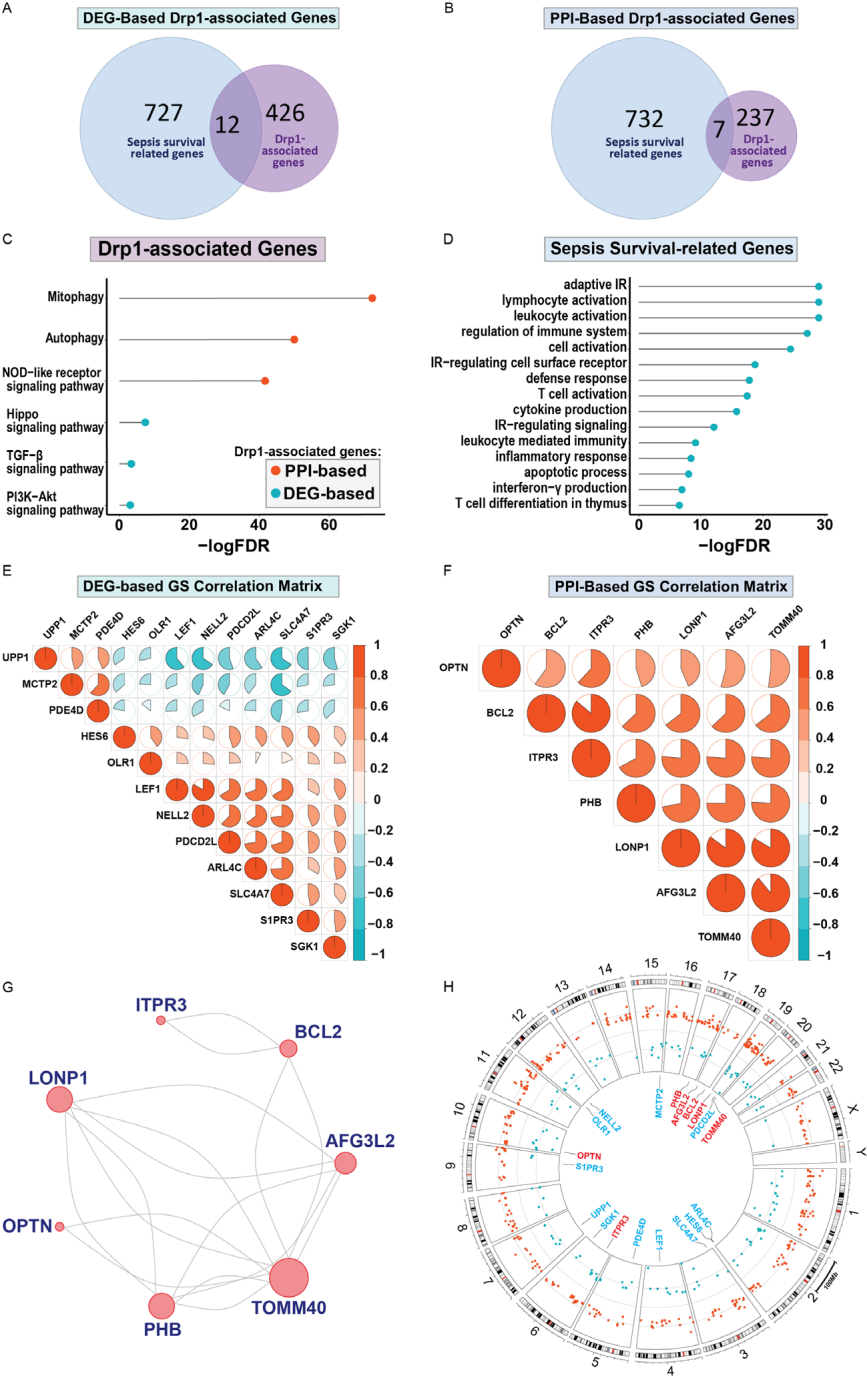
Differentially expressed genes and protein-protein interaction analysis revealed a 12 and 7 Drp1-associated gene signature that corresponds with sepsis survival genes. 12 of 438 Drp1-associated genes (generated by identifying DEG after Drp1 overexpression) match genes related to sepsis survival **(A)**, these 12 genes are referred to as DEG-based GS. PPI analysis of Drp1 identified 244 Drp1-associated genes. 7 of these genes are common between the Drp1-associated genes and sepsis survival genes **(B)**, these 7 genes are referred to as PPI-based GS. KEGG analysis was used to identify pathways enriched with both Drp1-associated GS **(C)** and pathways enriched with the sepsis survival genes **(D)**. The correlation matrix of Drp1-DGEs **(E)** and Drp1-PPI GS **(F)** shows the relationship of individual genes compared to the other genes in the GS. PPI network for the genes in the PPI-based GS **(G)**, and DEG-based GS have no interactions. Genomic locations for both GS are shown in a circular plot **(H)**, with the DEG-based GS represented by blue lettering, and the PPI-based GS represented by red lettering. Red dots represent upregulated genes and blue dots represent downregulated genes related to sepsis survival. Drp1, Dynamin-related protein 1; DEGs, differentially expressed genes; PPI, protein-protein interaction; KEGG, Kyoto Encyclopedia of Genes and Genomes; FDR, adjusted p-value; GS, gene signature; IR, immune response.

First, we sought to identify genes related to Drp1 protein expression. To accomplish this, we used the publicly available GEO dataset containing the gene expression data from human cells where Drp1 was overexpressed and from control cells (GSE182710). With this, we identified 438 genes differentially expressed (DEGs) after Drp1 overexpression, henceforth referred to as the DEG-based Drp1-associated genes (**Supplementary Table 1**). Next, we performed a protein-protein interaction (PPI) analysis of the protein, Drp1, to identify proteins predicted to interact with Drp1 using the STRING database. This method resulted in an additional 244 genes associated with Drp1, designated as PPI-based Drp1-associated genes (**Supplementary Table 2**). We then enriched these two sets of Drp1-associated genes with genes related to sepsis survival. This revealed 12 and 7 common genes between the sepsis survival genes and the DEG- or PPI-based Drp1-associated gene sets, respectively (**Figures 2A, B**, **Table 1**). We designated the 12 and 7 gene sets as DEGs and PPI Drp1-gene signatures (GS), respectively.

**Table 1:** List of genes in the Drp1-associated gene signatures.

Drp1-associated Genes	
GS Name	Gene
DEG-based GS	SLC4A7
	PDCD2L
	UPP1
	ARL4C
	NELL2
	LEF1
	SGK1
	PDE4D
	S1PR3
	MCTP2
	HES6
	OLR1
PPI-based GS	AFG3L2
	BCL2
	ITPR3
	LONP1
	OPTN
	PHB
	TOMM40

Two gene signatures were generated: Drp1-DGEs GS and Drp1-PPI GS. Drp1, Dynamin-related protein 1; DEGs, differentially expressed genes; PPI, protein-protein interaction; GS, gene signature.

We then performed KEGG pathway analysis using both the Drp1 DEGs and PPI gene signatures (**Figure 2C**). This revealed 6 pathways that are enriched in these genes: mitophagy, autophagy, the NOD-like receptor, TGF-β, and PI3K-Akt signaling pathways. We have previously published a gene signature that differentiates between patients with a low or high risk of sepsis-related death and compiled a list of genes related to sepsis survival (32). KEGG analysis of these genes reveals 15 pathways enriched in sepsis-survival genes, consisting of primarily immunomodulatory pathways (**Figure 2D**). We then assessed the correlation of the Drp1-associated genes with the other genes in their respective GS to validate that PPI GS was generated correctly (**Figures 2E, F**). As expected, the Drp1-DEGs GS consists of genes with relatively low correlation with one another (**Figure 2E**). On the other hand, the Drp1-PPI GS consists of genes that are highly correlated with one another (**Figure 2F**). The PPI network of this GS is shown in **Figure 2G**, meanwhile, no connection was found within Drp1-DEGs GS. The location of the genes of both GS are shown in **Figure 2H**, with the Drp1-DEGs GS represented by blue lettering, and the Drp1-PPI GS represented by red lettering in the inner circle. The dots in the middle circle represent the locations of genes related to sepsis survival, with red dots representing upregulated and blue dots representing downregulated sepsis survival-related genes.

**Figure 3:**
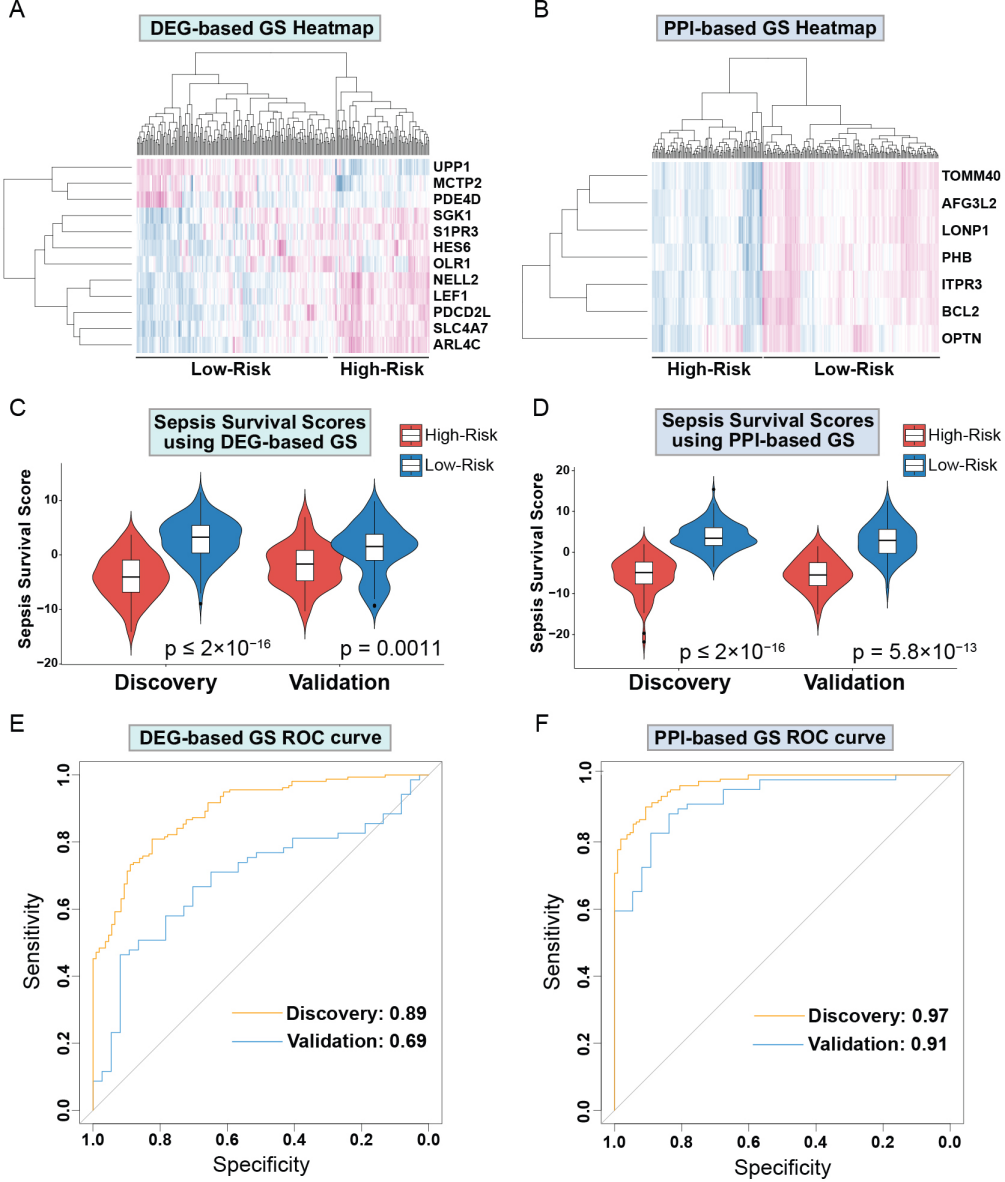
Drp1-associated gene signatures differentiate between low and high-risk sepsis groups in both the discovery and validation cohort. Heatmap of the DEG-based **(A)** and PPI-based GS **(B)** reveal expression patterns that differentiate low and high-risk sepsis groups. Violin plots of the sepsis survival scores from DEG-based **(C)** and the PPI-based **(D)** GS show that these gene signatures successfully differentiate between low and high-risk sepsis patients in both the discovery and validation cohorts. ROC curves of the DEG-based **(E)** and PPI-based **(F)** GS exhibit the sensitivity and specificity of these gene signatures. Drp1, Dynamin-related protein 1; DEGs, differentially expressed genes; PPI, protein-protein interaction; GS, gene signature; ROC, receiver operating characteristic curve.

**Figure 4:**
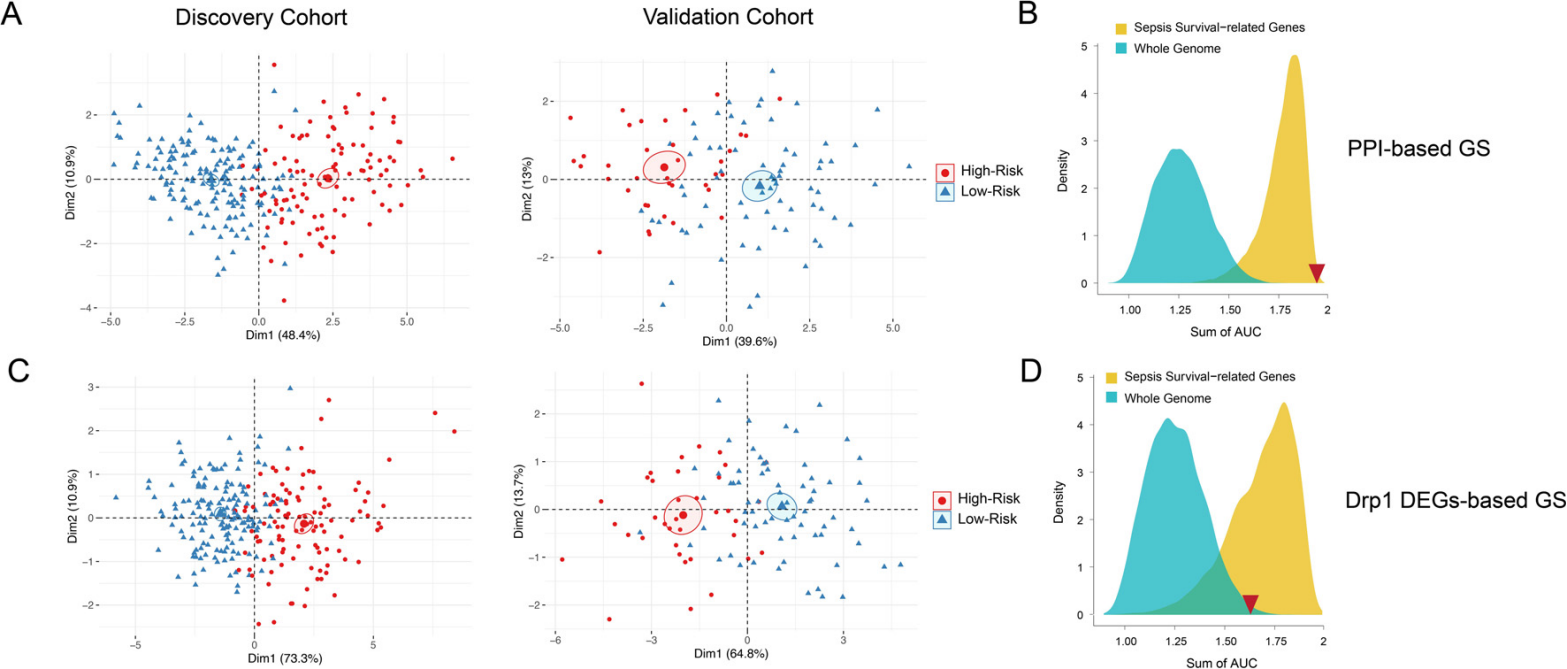
PCA and density distribution plots of both Drp1-associated gene signatures. PCA of both DEG-based **(A)** and PPI-based **(C)** GS were performed to reduce dimensionality and assess the similarity between each individual sample. The PCA showed that both gene signatures can entirely differentiate high-risk sepsis patients and low-risk sepsis patients. The PCA analysis for DEG-based GS represents 78.5-84.2% of the variable expression data. The PCA analysis for PPI-based GS represents 52.6-59.3% of the variable expression data. The density distribution of the AUC of random genes (blue) and sepsis survival-related genes (yellow) shows that both DEG-based GS **(B)** and PPI-based GS **(D)** have a prognostic power greater than random genes from the whole genome. The PPI-based GS **(D)** prognostic power is greater than that of other random genes related to sepsis survival. The sum of the DEG-based GS AUC value (1.58) and the sum of the PPI-based GS AUC value (1.88) is indicated by the red inverted triangles. Drp1, Dynamin-related protein 1; DEGs, differentially expressed genes; PPI, protein-protein interaction; GS, gene signature; PCA, principal component analysis; AUC, area under the curve.

Next, we sought to evaluate both GS for their capabilities of distinguishing high and low sepsis risk groups. To accomplish this, we first compiled the expression levels of the individual genes within the GS and separated the low and high-risk patients. We obtained heatmaps of the DEGs-based (**Figure 3A**) and PPI-based (**Figure 3B**) GS, which both show expression patterns that differentiate low and high-risk sepsis groups from the discovery cohort. To confirm this differentiation, we generated violin plots of the sepsis survival scores of both gene signatures in the discovery and validation cohorts. To calculate sepsis survival scores, we used our previously published formula (32), which assesses the probability of septic death in each patient. Lower score values indicate a worse prognosis and survival rate, we labeled this group as the high-risk septic patients. Higher scores were classified as low-risk septic patients. Both sepsis survival score violin plots demonstrate that the DEG-based (**Figure 3C**) and the PPI-based (**Figure 3D**) GS successfully differentiate between low and high-risk sepsis patients, with the PPI-based GS providing greater separation between the low and high-risk groups. To evaluate both the specificity and sensitivity of the gene signatures, we generated a receiver operating characteristic curve (ROC) for the DEG-based (**Figure 3E**) and PPI-based (**Figure 3F**) GS. These ROC curves reveal an AUC value of 0.89 for the discovery cohort using the DEG-based GS and 0.97 using the PPI-based GS. For the validation cohort, the DEG-based GS has an AUC value of 0.69 and the PPI-based GS has a value of 0.91, indicating that the PPI-based GS is more specific and sensitive compared to the DEG-based GS. Principal component analysis (PCA) of both gene signatures shows that the DEG-based (**Figure 4A**) and PPI-based (**Figure 4C**) GS can completely differentiate high-risk sepsis patients from low-risk patients in both the discovery and validation cohorts. The PCA analysis for DEG-based GS represents 78.5-84.2% of the variable expression data. The PCA analysis for PPI-based GS represents 52.6-59.3% of the variable expression data.

To confirm that both gene signatures are more predictive of sepsis risk than a random set of whole genome genes (blue) or a random set of sepsis-survival genes (yellow), we plotted the density distribution of the AUC for the whole genome and sepsis-survival genes (**Figures 4B, D**). The sum of the DEG-based GS AUC value (1.58) and the sum of the Drp1-PPI GS AUC value (1.88) are indicated by the red inverted triangles. These results indicate that both the DEG-based and PPI-based GS have a prognostic power greater than random genes from the whole genome. However, the PPI-based GS has prognostic power that is greater than that of other random genes related to sepsis survival, while the DEG-based GS does not. Therefore, we selected the PPI-based GS to calculate sepsis survival scores for the patients in the discovery cohort.

**Figure 5:**
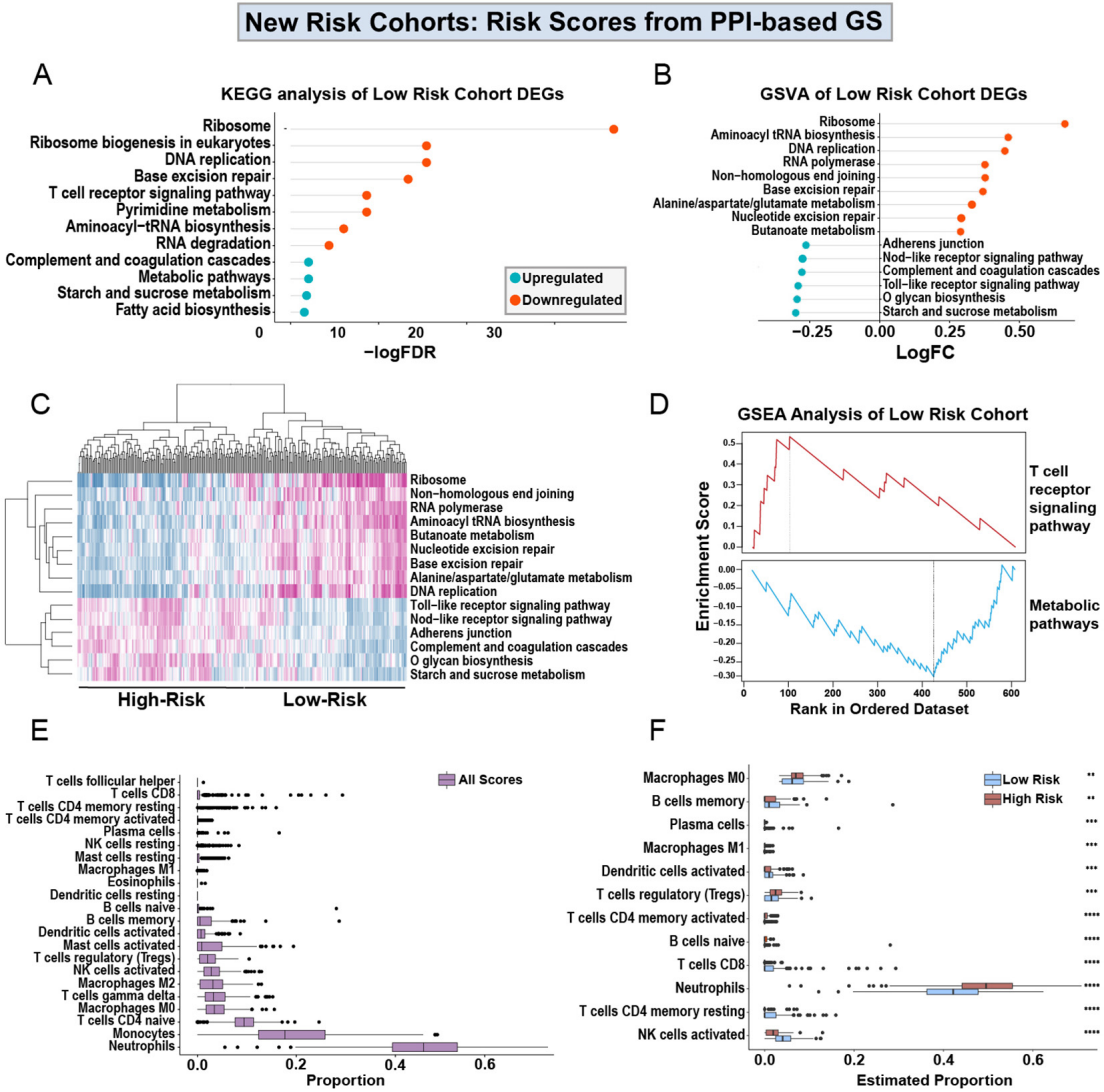
Analysis of low- and high-scores obtained from PPI-based GS. KEGG analysis of the upregulated and downregulated DEGs from high-score, lower-risk patients in the discovery cohort **(A)**, using the Drp1-PPI GS to generate the sepsis survival scores (**Supplementary Tables 3**, **4**). Gene set variation analysis (GSVA) shows the logFC of the pathways enriched in the DEGs from the high-score, lower-risk cohort **(B)**. The heatmap generated from the GSVA shows pathway expression patterns that differentiate the low- and high-score cohorts **(C)**. GSEA shows that the high-score, lower-risk cohort has increased T cell receptor signaling and decreased expression of metabolic pathways **(D)**. Using CIBERSORT, immune cell proportions in PBMCs were estimated in the entire discovery cohort **(E)**. Separating the low- and high-risk cohorts shows a predicted immune profile shift between the groups **(F)**. Specifically, the high-score, lower-risk cohort has decreased levels of neutrophils and increased activation of NK cells. Drp1, Dynamin-related protein 1; DEGs, differentially expressed genes; PPI, protein-protein interaction; KEGG, Kyoto Encyclopedia of Genes and Genomes; FDR, adjusted p-value; FC, fold change; GS, gene signature; GSVA, gene set variation analysis; GSEA, gene set enrichment analysis (GSEA).

In order to understand the cellular and molecular mechanism of the prognostic power of the PPI-GS, we compared the transcriptomic and cellularity differences between the high-score and low-score groups. Using the PPI-based GS, we then re-clustered the patients into high-survival score (**Supplementary Table 3**) or low-survival score (**Supplementary Table 4**) cohorts based on whether the survival scores of these samples were higher or lower than the medium survival score across all samples. With the new cohorts generated from the PPI-based GS, we identified DEGs in the high-survival score cohorts in comparison to the low-survival score cohorts, where low-survival scores indicate a higher-risk group for sepsis severity. KEGG analysis shows that the upregulated genes in the high-score, low-risk cohort are enriched in the ribosome, DNA replication, T cell, and RNA pathways. While the downregulated genes in this lower-risk group are enriched in the metabolic and the complement and coagulation pathways (**Figure 5A**). We then utilized Gene Set Variation Analysis (GSVA) to identify additional pathways enriched in the high scores, lower-risk cohort (**Figure 5B**). This is highly consistent with the KEGG analysis in DEGs. We then generated a heatmap of the pathways identified by GSVA. This revealed pathway enrichment patterns that could differentiate between low- and high-sepsis survival score patients (**Figure 5C**).

Using gene set enrichment analysis (GSEA), we found that the high-score, lower-risk cohort has increased T-cell signaling and decreased metabolic signaling (**Figure 5D**). Because the results from KEGG, GSVA, and GSEA all indicate an alteration in immune pathways (**Figures 5A–D**), we used CIBERSORT to estimate the proportions of immune cells in the entire discovery cohort (**Figure 5E**). Lastly, we separated the low- and high-risk cohorts from one another (**Figure 5F**). This separation reveals a predicted immune profile shift between the low and high-risk groups. Specifically, the lower-risk patients are predicted to have decreased levels of neutrophils and increased activation of NK cells.

## 4 Discussion

Despite immense research efforts, there are still no available therapeutics for treating sepsis other than general antibiotics and source control. Early recognition and action are the only courses of action for physicians to improve patient outcomes (12, 36). This necessitates a quick diagnosis to reduce sepsis mortality. Unfortunately, the identification of patients at risk of septic death may not be sufficient to reduce mortality rates. This is exemplified in a retrospective cohort study of 6 hospitals between 2014 and 2015, where only 3.7% of sepsis-associated deaths were considered to be either moderately or definitively preventable (37). This suggests that even early identification of high-risk patients and subsequent implementation of aggressive treatments will only minimally improve sepsis outcomes. This highlights the urgency to identify new strategies to test and implement to improve sepsis survival. In this study, we identified Drp1-associated genes as a predictor of sepsis survival, which not only reveals a novel biomarker but also calls attention to mitochondrial dynamics as a pathway that should be investigated for therapeutic development.

Recent research efforts have called attention to the potential and likely connection between septic organ failure and mitochondrial function (27, 38). For example, it has been proposed that mitochondrial dysfunction may explain why there is minimal cell death observed during sepsis-induced organ failure. Additionally, mitochondrial dysfunction could also explain how tissues maintain oxygen levels during sepsis. Moreover, mitochondria have been speculated to mediate the rapid recovery of organ function in survivors, even in organs notoriously poor at regeneration (39–43). Since mitochondria are the leading harnessers of oxygen, it is plausible that mitochondrial function may explain these seemingly paradoxical findings. At the center of mitochondrial function is mitochondrial dynamics, in which networks of mitochondria alter their shape to modulate their function. Importantly, mitochondrial dynamics connects a multitude of pathways (23) and has vast implications for diseases with multiple perturbed pathways, including sepsis. Here, we provide evidence that mitochondrial dynamics is connected to sepsis severity by demonstrating the prognostic capability of the major regulator of fission, Drp1. Additionally, we demonstrate that Drp1 and its associated proteins likely influence multiple signaling pathways.

Historically, sepsis pathobiology was credited to excessive inflammation. However, this is now widely disputed, resulting in sepsis being redefined (1). It is now accepted that both pro- and anti-inflammatory responses are activated during the early phases of sepsis (17) and that a multitude of nonimmunological pathways are perturbed in addition to abnormal immune responses (44–46). Activation of the Toll-Like Receptor 4 (TLR4) signaling pathway is regarded as a critical component of sepsis (47), releasing pro-inflammatory mediators and reactive oxygen and nitrogen species (48). Attempts to antagonize TLR4 have failed to improve mortality rates (16), suggesting that TLR4 activation may contribute to sepsis development; however, severity may be determined by a more downstream component. In support of this, we recently published that TLR4 agonists increase mitochondrial fission through activation of Drp1 (49). This and our previously published findings lead us to conjecture that mitochondrial dynamics play a critical role in determining sepsis severity. It is tempting to speculate that pathways activated during sepsis may modulate mitochondrial dynamics, leading to dysfunction that perpetuates mitochondrial damage and disease progression. Postulations aside, this study demonstrates that Drp1-associated proteins can predict sepsis severity, implicating mitochondrial dynamics in sepsis progression.

Interestingly, the PPI-based GS predicted sepsis survival better than the DEG-based GS, as indicated by higher specificity and sensitivity values. A plausible explanation for this is that the PPI-based GS consists of proteins that interact with Drp1. Therefore, we may be identifying genes that have more functional outcomes than genes identified by Drp1 overexpression. Although further testing is needed to evaluate this, it does support further evaluation of mitochondrial dynamics during sepsis. In this study, we used the STRING database to identify proteins that interact with Drp1, which generates functional protein association networks grouped into shells. In our analysis, we only used the primary protein interactors. The primary interactors represent proteins that are directly associated with Drp1. On the other hand, the secondary protein interactors (or the 2^nd^ shell) contain proteins that associate with primary protein interactors. In this study, we hypothesized that mitochondrial dynamics are capable of predicting sepsis survival. For that reason, we evaluated the expression of proteins that are directly associated with Drp1. It would be interesting to perform further analysis using the 2^nd^ shell to elucidate which proteins may be enhancing or inhibiting Drp1 activity. Additionally, it would be of value to identify specific pathways these 2^nd^ shell proteins interact with to isolate a gene signature with even greater prognostic ability.

In this study, we used datasets from two different biological sources: primary human peripheral blood mononuclear cells (PBMC) and human cell lines. Because the aim of this study was to evaluate the effects of altered mitochondrial dynamics on sepsis survival, we chose to focus our study on the major fission regulator, Drp1, and proteins that are directly affected by Drp1. To identify genes directly related to Drp1, human cell lines are necessary, as Drp1 deletion causes embryonic lethality in mice (50). Moreover, by using data from cells, we are only gathering genes directly affected by Drp1 upregulation. Global upregulation of Drp1 in mice causes immense damage, which would convolute our analysis and cause such a high degree of dysfunction that the resulting data would no longer have biological applicability to sepsis (51–53). We then used existing PBMC expression data to confirm that these genes are detectable and capable of differentiating between low- and high-risk patients. By using PBMC from septic patients, we validated that the identified genes derived from the human cell lines are relevant in a clinical setting.

One major limitation of this study is the low number of septic patients with available genetic data. Small data sets are inherently more prone to bias that may alter or even hide important genetic evidence. In our study, we used the data set E-MTAB-4421 as the discovery cohort, consisting of 45.6% female samples, while the validation cohort E-MTAB-4451 has only 26.3% female samples. Sex biases in medicine and research are well documented (54–59), and sex disparity in sepsis severity and survival is also noted in the field (60). An analysis of sepsis data from 2017 indicates that the global incidence of sepsis is higher among females (61). This presents a critical unmet need for studying the sex differences in the response to sepsis and highlights the necessity to specifically study sepsis and sex, and include pregnancy status (62, 63). Further, sepsis is well recognized as a heterogeneous disease state (64), meaning that the pathogenesis of sepsis is highly influenced by the pathogen, site of infection, co-morbidities, race, and sex (65–69). Therefore, the current transcriptome databases are substantially inadequate, and major effort should be allocated to obtaining larger datasets that include information on all of these factors for identification of the expression differences between groups that may be hidden by aggregating all the different groups together. Additionally, these datasets lack detailed patient information and the treatments they received. Because of this lack of information, there may be confounding factors between the patients who survive and those who do not. Despite these limitations, we still believe GS could be a valuable tool to identify high-risk patients.

**Figure 6:**
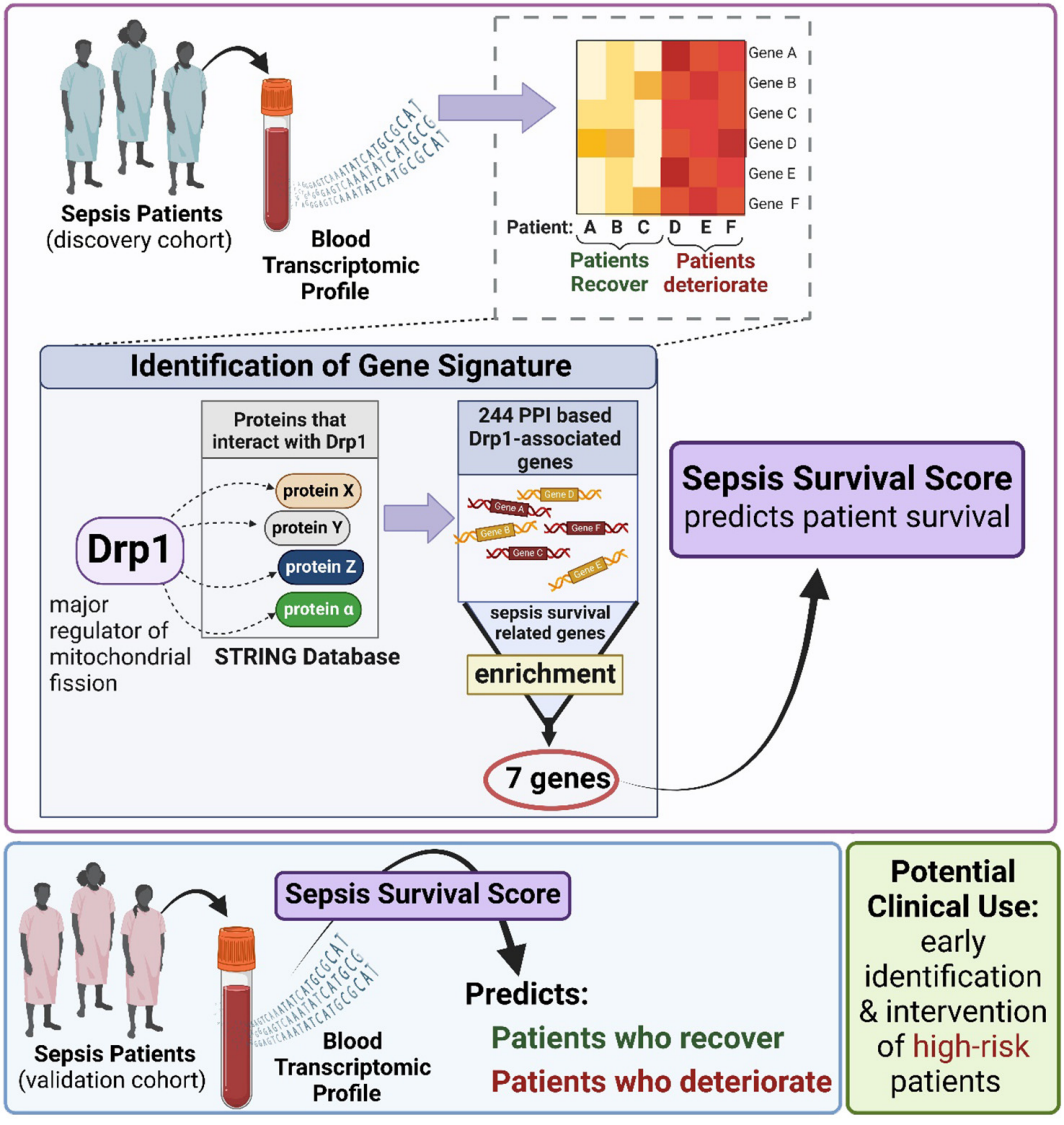
Gene signature was generated using patient plasma samples to predict sepsis prognosis.

Another consideration while interpreting the results from our study is that the generation of this GS was a computational analysis using data from a retrospective study. Therefore, to confirm our findings, a prospective study is needed where GS is used at ICU admission to determine if the high-risk group identified by the GS has a higher mean length of ICU stay or mortality rate than the low-risk group. If successful, this may lead to the development of precision medicine for this group that counters this expression pattern. Alternatively, this could provide an opportunity to test therapeutics that have previously failed to demonstrate significance in clinical trials. Specifically, if our hypothesis of mitochondrial dynamics being a critical mediator of sepsis severity is correct, that could mean that drug candidates were tested in a population with too diverse of sepsis presentations. Sepsis consists of many perturbed pathways that interact with one another and with a person’s specific genetics or comorbidities. Thus, an individual’s presentation of sepsis can vary. Therefore, identifying high-risk patients with GS could identify a sub-population that would be responsive to therapies that target mitochondrial dynamics. Although more testing is needed to confirm the validity of those speculations, it is clear that the early evidence we provide here provides support that this generated gene signature may provide novel clinical opportunities shown in **Figure 6**.

This current gene signature, can propel the field forward in understanding the complexity that is sepsis and understand the clinical implications of mitochondrial health. Moreover, as expected, this gene signature might be applied to other chronic and acute diseases due to its nature as a composite biomarker (70–73). Mitochondrial dysfunction is closely associated with a variety of diseases, including SARS-CoV-2 and cancer (74–77). However, it is essential to recognize that the gene signature will be disease-specific and will require careful adjustment and validation for each specific condition. This aligns with the principles of precision medicine. In addition, it is important to recognize that many challenges exist in translating results from bioinformatic analyses into the clinic. For example, one significant challenge is mitigating errors during computational analysis. Overfitting during bioinformatics analyses can lead to false-positive results, making the gene signature cohort-specific and lacking the precision and accuracy needed for reliable disease prognosis in independent cohorts. To address this, two critical steps should be employed: (a) validation in independent cohorts and (b) comparison of the gene signature’s performance against randomized gene signatures, as illustrated in our **Figure 3**. However, even while taking these precautions, it is essential that gene signatures are tested clinically before widespread use.

In summary, septic organ dysfunction, regardless of severity, is not associated with cell death (41). This suggests that sepsis involves changes in functional signaling rather than organ cell death. A leading theory is that cells are unable to maintain homeostasis during sepsis (46). Specifically, cells are unable to counteract perturbed pathways to return to normal physiological function. Mitochondria are known to play critical roles in maintaining cellular homeostasis (22) and are implicated in sepsis (25–28). Therefore, mitochondria may present an opportunity to restore multiple dysregulated pathways using a single strategy. In this study, we demonstrate that genes related to the major mitochondrial shaping protein, Drp1, can predict sepsis severity. For that reason, mitochondrial dynamics not only offers a prognostic opportunity but also suggests a novel therapeutic strategy that should be investigated further.

## 5 Conclusions

By engineering and validating gene signatures linked to Drp1, we demonstrated that peripheral blood gene expression can accurately predict sepsis survival. With further studies, this innovative sepsis prognostic biomarker (Drp1-PPI GS) can be developed for clinical application.

## Data Availability

The original contributions presented in the study are included in the article/**Supplementary Material**. Further inquiries can be directed to the corresponding author.
